# Generation of Eroded Nanoplastics from Domestic Wastes and Their Impact on Macrophage Cell Viability and Gene Expression

**DOI:** 10.3390/molecules29092033

**Published:** 2024-04-28

**Authors:** Mohammad Saiful Islam, Indrani Gupta, Li Xia, Arjun Pitchai, Jonathan Shannahan, Somenath Mitra

**Affiliations:** 1 Department of Chemistry and Environmental Science, New Jersey Institute of Technology, Newark, NJ 07102, USA; mi238@njit.edu (M.S.I.); ig72@njit.edu (I.G.); 2 School of Health Sciences, Purdue University, West Lafayette, IN 47907, USA; xia104@purdue.edu (L.X.); apitchai@purdue.edu (A.P.); jshannah@purdue.edu (J.S.)

**Keywords:** microplastics, nanoplastics, top-down milling technique, machrophage cell viability, inflammatory gene expression

## Abstract

This study reports an innovative approach for producing nanoplastics (NP) from various types of domestic waste plastics without the use of chemicals. The plastic materials used included water bottles, styrofoam plates, milk bottles, centrifuge tubes, to-go food boxes, and plastic bags, comprising polyethylene terephthalate (PET), polystyrene (PS), polypropylene (PP), high-density polyethylene (HDPE), and Poly (Ethylene-co-Methacrylic Acid) (PEMA). The chemical composition of these plastics was confirmed using Raman and FTIR spectroscopy, and they were found to have irregular shapes. The resulting NP particles ranged from 50 to 400 nm in size and demonstrated relative stability when suspended in water. To assess their impact, the study investigated the effects of these NP particulates on cell viability and the expression of genes involved in inflammation and oxidative stress using a macrophage cell line. The findings revealed that all types of NP reduced cell viability in a concentration-dependent manner. Notably, PS, HDPE, and PP induced significant reductions in cell viability at lower concentrations, compared to PEMA and PET. Moreover, exposure to NP led to differential alterations in the expression of inflammatory genes in the macrophage cell line. Overall, this study presents a viable method for producing NP from waste materials that closely resemble real-world NP. Furthermore, the toxicity studies demonstrated distinct cellular responses based on the composition of the NP, shedding light on the potential environmental and health impacts of these particles.

## 1. Introduction

An estimated 9 to 23 million metric tons of plastic are discharged into rivers, lakes, and oceans every year, a considerable portion of which ultimately transforms into microplastics (MPs) and nanoplastics (NPs). Currently, there are approximately 0.3 million metric tons of plastic floating on the ocean’s surface [1,2,3,4]. Studies conducted on mouse models have shown that ingested plastic particles can be detected in tissues outside of the gastrointestinal tract, including the liver, kidney, brain, and circulation [5]. Recently, measurable quantities of NPs were detected in the blood of 77% of human volunteers [6]. Safety assessments of microplastics in mouse models have revealed acute toxicity, including inflammation, oxidative stress, and lipid dysregulation, which corresponded to changes in plastic particle size, concentration, and tissue accumulation. Comparable toxicity responses were also observed in human cell culture models [7]. Gene expression, microbiota composition, behavioral impairments, inflammatory and oxidative stress-related markers, and pathological changes in major organs of a mouse model have also been observed [8]. Fetal mice exposed to inhaled microplastics have also displayed systemic toxicity. Administration of polyethylene microplastics to pregnant dams through intratracheal instillation has also exhibited toxicity [9].

The study of nanoplastics (NP) has posed several challenges, due to the diverse range of materials falling under this category [10]. Additionally, the size and shape of NPs have been found to play a significant role in their uptake, adsorption, and biological effects [10,11]. Microplastics (MP) are defined as having a size between 100 nm–5 mm, while nanoplastics (NP) are less than 100 nm [12]. Microplastics have been identified in ecological samples, demonstrating their environmental presence [13]. There is a recognized potential for plastic particulates to have human health impacts and a need for toxicological evaluation of human-relevant exposures [14,15]. In the context of toxicity studies, most research on MP and NPs has relied on commercially available synthesized polystyrenes, produced using methods like emulsion polymerization [16,17,18,19]. However, these pristine MP/NPs, characterized by their uniform shape and size, differ substantially from real-world plastics, lacking the additives present in actual plastic products. As a result, there is a scarcity of research on the toxicity of MP/NPs derived from real-world plastic materials on cells. To address this knowledge gap, studies utilizing these real-world materials are essential [17].

To obtain more precise measurements of the toxicological impacts of MP/NP, it becomes imperative to synthesize them from real-world plastic waste. Several methods have been employed to create MPs and some NPs for use as additives in polymer composites and adsorbents. These methods include cryogenic milling [16], centrifugal milling with liquid nitrogen [20], emulsion polymerization [21,22], laser ablation [23], and ultrasonication under strong basic conditions [24]. To produce NPs from real-world macro and microplastics, a solubilizing agent is added to plastic mixtures, followed by rapid vortexing and extraction of the organic solvent [25]. However, it is important to note that the use of cryogenics and organic solvents may alter the morphology, crystallinity, and other properties of the plastic, potentially affecting its nature. Therefore, for conducting accurate toxicity studies, it becomes essential to synthesize NPs using methods that preserve the chemical and physical properties of these particles. By doing so, researchers can gain more reliable insights into the toxicological effects of MP/NP on various systems and organisms.

The objective of this project was two-fold: first, to synthesize NP from real-world plastic waste while maintaining their chemical and crystal structures, without using chemicals or cryogenics. Second, to investigate the impact of these particles on macrophage cell viability and the expression of genes associated with inflammation and oxidative stress. 

## 2. Results and Discussion

### 2.1. Sample Characterization

**Figure 1:**
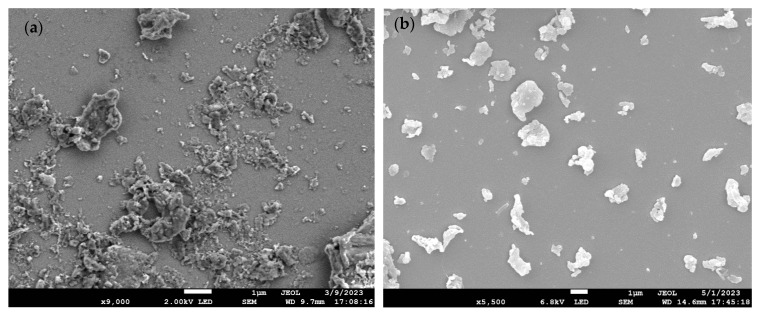

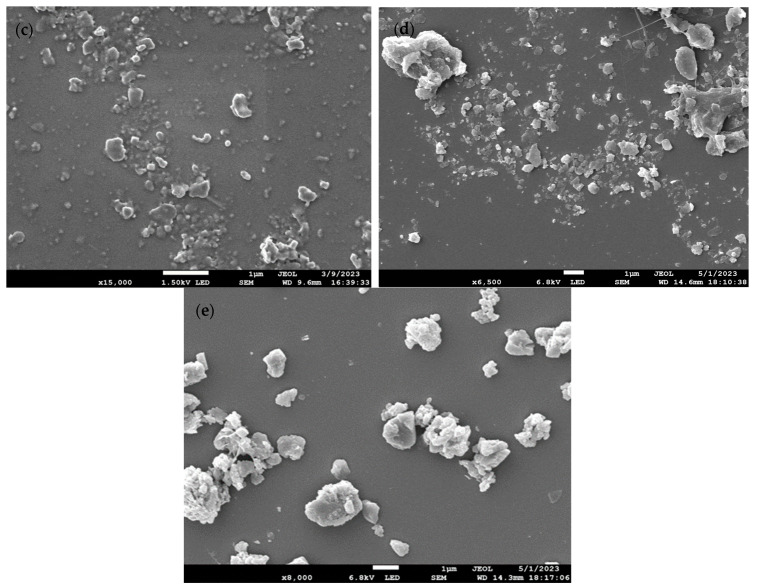
SEM images of (**a**) PET-W, (**b**) PS-P, (**c**) HDPE-M, (**d**) PP-C, and (**e**) PEMA-5.

Figure 1a–e shows the scanning electron microscope (SEM) images of PET, PS, HDPE, PP, and PEMA, respectively. As evident from Table 1, the milling process proved successful in generating MP and NP, confirming the effective milling of the raw materials. As anticipated, the particles exhibited irregular shapes and displayed a size distribution characteristic of real-world weathering and abrasion processes. The results align with expectations, further validating the production of MP/NP particles through the milling method.

After the milling process, the particles appeared as agglomerates. To separate these agglomerations and isolate the nanosized particles, sonication was employed. The resulting NP were then characterized, and their particle sizes, zeta potential, and polydispersity index (PDI) when suspended in water are listed in Table 1. Upon deagglomeration, the NP showed smaller sizes, compared to the original milled MPs. The analysis of the NPs revealed that the lowest particle size was observed for PET particles, with a mean diameter of 50.75 nm and a polydispersity index (PDI) value of 0.075. Following PET, HDPE particles exhibited a mean average particle size of 141.8 nm, with a PDI value of 0.558. On the other hand, Styrofoam plate-derived plastic ingredients (PS) showed significantly larger nanosized particles, approximately 396.1 nm in diameter. Similarly, centrifuge-extracted plastic ingredients from polypropylene (PP) exhibited a larger particle size in the nano range, approximately 255 nm. The PDI data for all the plastic samples indicated a larger size distribution, except for PET, which displayed a lower PDI value, suggesting a more uniform size distribution. In terms of stability, the zeta potential analysis showed that all the NPs exhibited good stability, with zeta potential values ranging from −22.4 mV to −32.1 mV. Overall, the findings confirm the successful generation of NP with varying sizes, stability, and size distributions, depending on the plastic material used. 

**Figure 2:**
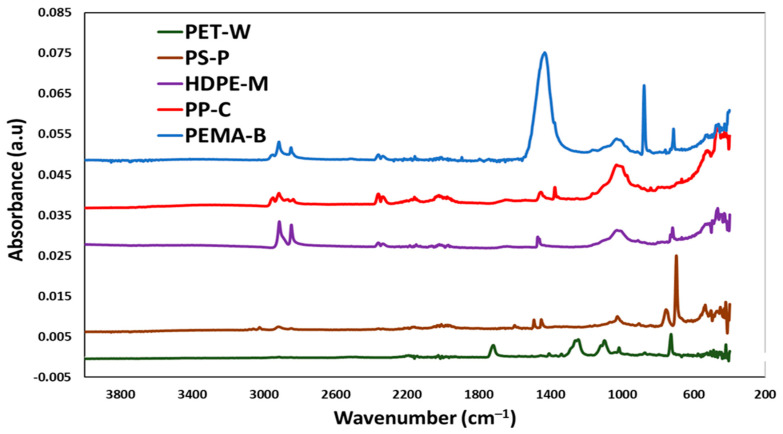
FTIR analysis of the synthesized nanoplastics (NP).

The FTIR analysis of the plastic materials is shown in Figure 2. Plastic 1 (PET-W) exhibited its distinctive peaks at 1712 cm^−1^, which is the C=O ester group, and the C-H out-of-plane deformation of two carbonyl substituents on the aromatic ring is observed at 730 cm^−1^ [20,21,22]. The two peaks at 1340 and 1150 cm^−1^ are attributed to -CH2- deformation band and C(O)-O stretching of ester groups, respectively [26]. Typical Plastic 2 (PS-P) absorption bands, at around 698, 757, 1029, 1160, 1350, 1494 and 1601 cm^−1^, can be seen clearly in the FTIR spectrum. Plastic 3 (HDPE-M) exhibited aliphatic C-H bands at 2915 and 2940 cm^−1^, and methyl bands at 1470 cm^−1^ [23]. The peak at 995 cm^−1^ in the FTIR spectrum of Plastic 4 (PP-C) is due to the vibration of the PP particle’s crystalline phase [25]. Pure Plastic 5 (PEMA-5) spectra has peaks at 2980 cm^−1^, which can be attributed to asymmetric C-H stretching and O-H stretching, and the O-CH_3_ ester group is represented by the peak at 1449 cm^−1^ [27]. 

**Figure 3:**
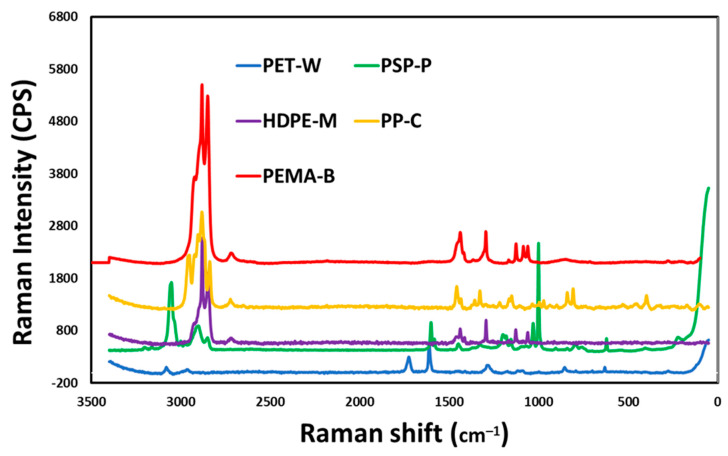
RAMAN analysis of the synthesized nanoplastics (NP).

Analysis of the Raman spectra collected from the samples are shown in Figure 3. Spectral features for PET-W show that the bands caused by the stretching frequencies of the C-H bonds are found at about 3000 cm^−1^; however, the maximum relative to the C=O bond and the maximum relative to the C-C bond in the aromatic ring are found at 1500 cm^−1^ and 1400 cm^−1^, respectively. For PS-P particles, the masses of the atoms involved, and the strength of their bonds, affect the vibration’s frequency. Low Raman shifts are found in heavy atoms and weak links. Strong bonds and light atoms have high Raman shifts. The PS-P spectrum exhibited high frequency carbon-hydrogen (C-H) vibrations at a wavelength of about 3000 cm^−1^. The wavelength of low frequency carbon-carbon (C-C) vibrations is 800 cm^−1^. Because hydrogen is lighter than carbon, C-H vibrations have a higher frequency than C-C vibrations. Two carbon atoms joined together by a stronger single bond (C-C) vibrate at about 800 cm^−1^, while two carbon atoms joined together by a double bond (C=C) vibrate at about 1500 cm^−1^. The Raman spectrum of HDPE-M shows the bands due to the stretching frequencies of the C-H bonds at about 3000 cm^−1^, the bending and twisting frequencies of the C-H bonds at 1300 cm^−1^ and about 1400 cm^−1^, and the stretching of the C-C bonds between 1000 and 1200 cm^−1^. The main bands of PP-C, due to the C-C, CH_3_ and CH_2_ bonds, are recognized at 1400 cm^−1^, 1100 cm^−1^ and 3000 cm^−1^. The Raman spectrum of PEMA-5 include the main bands, due to the C-C, C=C, CH_3_ and C-O bonds at 3000, are recognized.

**Figure 4:**
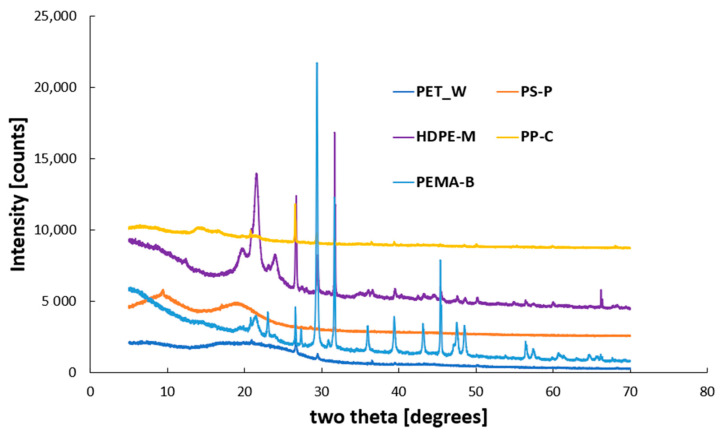
XRD analysis of the synthesized nanoplastics (NP).

The powder X-ray diffraction (XRD) analysis of synthesized nanoplastics (NP) presented in Figure 4 shows the sharp and intense crystalline peak intensity and demonstrates this crystallinity. Additionally, crystallite size for the nanoplastics are important properties. Based on the XRD HighSchore Plus (version 5.2) software analysis, we noticed that all the nanoplastics within the strong peak intensity at major peaks produce a smaller crystallite size of several 100 Å (Figures S1–S5).

### 2.2. Plastic Particulate-Induced Alterations in Macrophage Viability

**Figure 5:**
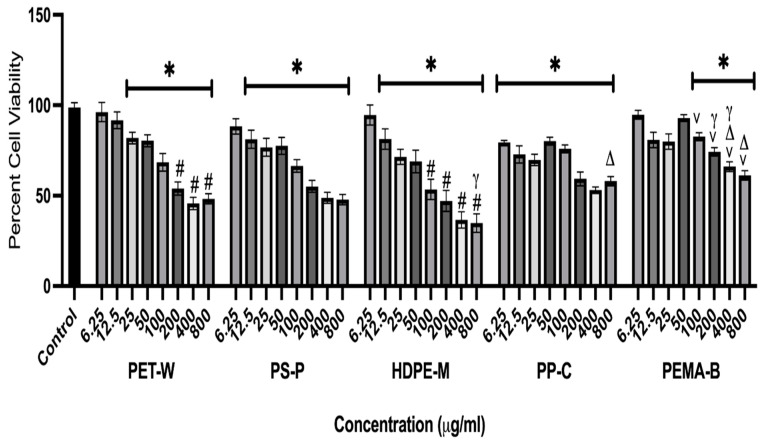
Cell viability was assessed following 24 h exposure to plastic particulates. Macrophages were exposed to PET-W, PS-P, HDPE-M, PP-C, or PEMA-B plastic particulates, at concentrations of 0 (control), 6.25, 12.5, 25, 50, 100, 200, 400, and 800 μg/mL for 24 h. Untreated cells served as controls for the MTT assay. * Denotes statistical significance compared to the control group, # denotes statistical significance compared to PEM-B at the same concentration, γ denotes statistical significance compared to PP-C at the same concentration, v denotes statistical significance compared to PS-P at the same concentration, and Δ denotes statistical significance compared to HDPE-M (*n* = 4/group, *p* < 0.05).

A macrophage cell line was exposed to PET-W, PS-P, HDPE-M, PP-C, and PEMA-B at concentrations of 0 (untreated control), 6.25, 12.5, 25, 50, 100, 200, 400, and 800 μg/mL for 24 h to determine differential exposure-induced cytotoxicity (Figure 5). Plastic particulate exposures induced concentration-dependent decreases in cell viability. 

All plastic particulates reduced cell viability; however, PS-P, HDPE-M, and PP-C induced significant reductions at lower concentrations, compared to PET-W and PEMA-B. PEMA-B did not induce significant reductions in cell viability until reaching a concentration of 100 μg/mL. A previous evaluation of PP NPs demonstrated significant cytotoxicity in human airway Type II epithelial cells (A549) at a concentration of 4 mg/mL after a 16 h exposure [26]. The current study demonstrated cytotoxicity at lower concentrations. Specifically, for the PP NPs, mouse macrophages demonstrated cytotoxicity at 6.25 μg/mL after 24 h of exposure. This differential in cell death may result from differences in cell susceptibility (human epithelial cells compared to mouse macrophage), PP NP size (660 vs. 255 nm), preparation method (milling compared to commercial PP particles chemically precipitated), and/or the suspension method (dimethyl sulfoxide compared to cell culture media supplemented with fetal bovine serum). 

In another evaluation of pristine PS NPs no cytotoxicity was observed in RAW 264.7 mouse macrophages at concentrations up to 100 μg/mL following a 24 h exposure [28]. However, addition of -COOH and -NH2 surface functionalization enhanced cytotoxicity, with significant cell death occurring at 20 and 1 μg/mL, respectively [28]. While this evaluation utilized the same macrophage model and exposure time point, it however differed, based on the utilization of commercially available uniform PS NP with a spherical size of 100 nm. The PS NPs utilized in our current study were larger (396.1 nm), had irregular shapes due to the production procedure, and demonstrated a more negative zeta potential. Further, in another toxicity evaluation of PS particulates using RAW 264.7 mouse macrophages, cell death was determined to be size dependent, with 80 nm PS NPs inducing greater cell death than 3 μm PS microplastics [29]. Specifically, significant cell death was determined at 0.1 μg/mL of 80 nm PS NPs and 5 μg/mL of 3 μm PS microplastics following a 24 h exposure. Our assessment demonstrated cell death for our PS NPs at concentration 12.5 μg/mL. Together, these findings suggest that when size decreases, the cytotoxicity of the PS particle increases. 

### 2.3. Evaluation of Plastic Particulate-Induced Differential Macrophage Inflammatory and Oxidative Stress Response

**Figure 6:**
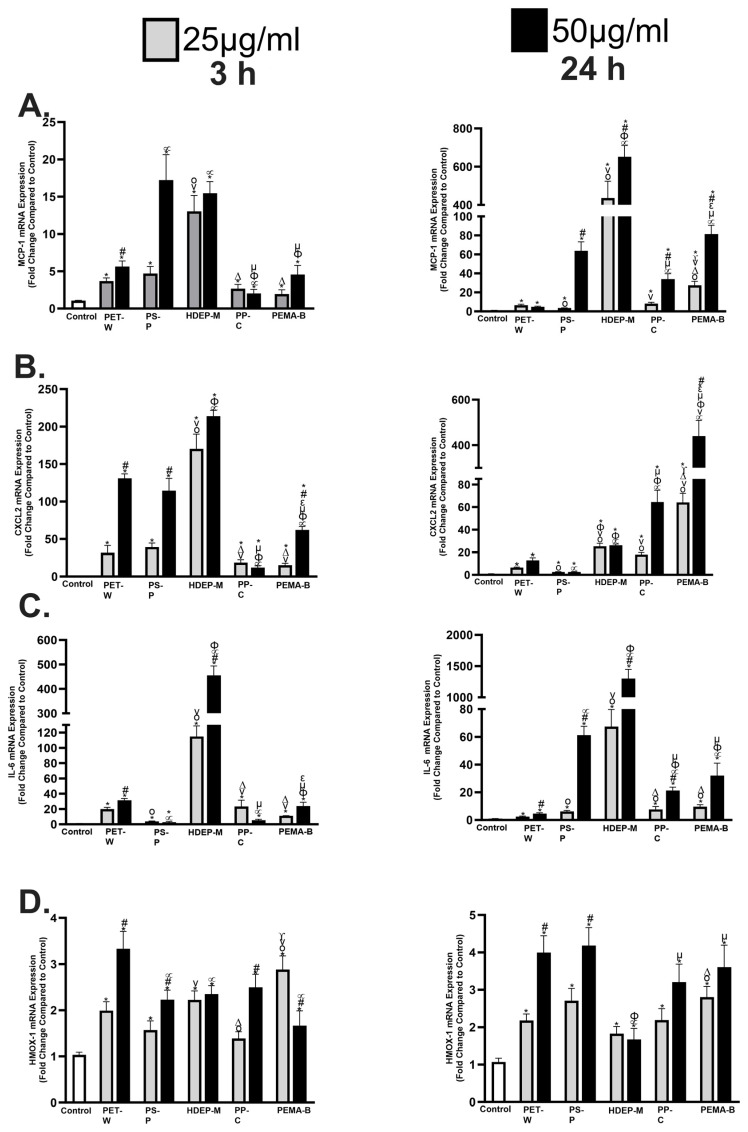
Alterations in gene expression of inflammatory and oxidative stress markers following plastic particulate exposure. Expression of mRNA was assessed relative to untreated controls. Macrophages were exposed to 25 or 50 μg/mL of PET-W, PS-P, HDPE-M, PP-C, or PEMA-B plastic particulates for 3 or 24 h. Gene expression of MCP-1 (**A**), CXCL2 (**B**), IL-6 (**C**), and HMOX-1 (**D**) and GAPDH (housekeeping gene) was assessed through real-time rtPCR to evaluate plastic particulate inflammatory responses and oxidative stress. * Denotes statistical significance compared to the expression of the control group, # Denotes statistical significance between concentrations of 25 and 50 μg/mL of the same plastic particulate, ° Denotes statistical significance compared to 25 μg/mL of PET-W, v Denotes statistical significance compared to 25 μg/mL of PS-P, Δ Denotes statistical significance compared to 25 μg/mL of HDPE-M, γ Denotes statistical significance compared to 25 μg/mL of PP-C, ∝ Denotes statistical significance compared to 50 μg/mL of PET-W, ϕ Denotes statistical significance compared to 50 μg/mL of PS-P, μ Denotes statistical significance compared to 50 μg/mL of HDPE-M, ε Denotes statistical significance compared to 50 μg/mL of PP-C. (*n* = 4/group, *p* < 0.05).

Inflammatory gene expression alterations were examined in a macrophage cell line following exposure to 25 or 50 μg/mL of PET-W, PS-P, HDPE-M, PP-C, or PEMA-B for 3 h or 24 h (Figure 6). Macrophage chemoattractant protein-1 (MCP-1), a marker of inflammation, was induced following exposure to all plastic particulates at both concentrations and time points (Figure 6A). Differential induction of MCP-1 was determined based on plastic particulate type. For example, at 3 h PP-C induced the least up-regulation of MCP-1 gene expression at both 25 and 50 μg/mL, while HDEP-M induced the greatest up-regulation at 25 μg/mL and PS-P and HDEP-M induced the greatest up-regulation at 50 μg/mL. At 24 h exposure to HDEP-M induced the greatest MCP-1 upregulation at both 25 and 50 μg/mL, while PET-W induced the least. 

Macrophage inflammatory protein-2 (CXCL2), another marker of inflammation, was induced following exposure to all plastic particulates at both concentrations and time points (Figure 6B). At 3 h, HDEP-M induced the greatest up-regulation of CXCL2, compared to other particles, whereas PP-C induced the least in both concentrations examined. At 24 h, PS-P induced the least up-regulation, while PEMA-B induced the greatest up-regulation for both concentrations, in terms of CXCL2 gene expression. Interleukin-6 (IL-6), a marker of inflammation, was up-regulated following all plastic particulate exposures at both concentrations and time points in the macrophage model (Figure 6C). At 3 h, PS-P induced the least up-regulation of IL-6 and HDEP-M induced the greatest up-regulation. At 24 h, PET-W demonstrated the least induction of IL-6 gene expression, whereas HDEP-M demonstrated the most up-regulation in both concentrations evaluated. In vivo pulmonary instillation studies have demonstrated PP NPs can induce a pulmonary inflammation within a mouse model via phosphorylation of NFκB and p-38 [28]. NFKB regulates the inflammatory genes evaluated in our assessment (CXCL2, MCP-1, and IL-6), suggesting the NPs may have stimulated macrophage inflammation via NFKB [30]. 

Interestingly, an in vitro evaluation of PS particles comparing 80 nm and 3 μm utilizing RAW 264.7 mouse macrophages demonstrated inductions of IL-6 and other inflammatory genes at concentrations lower than those examined in our current study [29]. Examination of these PS particles determined the induction of IL-6 gene expression at 24 h was size-dependent, with smaller NPs inducing a greater induction than larger microplastics. Heme-oxygenase-1 (HMOX-1), a marker of oxidative stress, was induced in macrophages following exposure to all plastic particulates at both concentrations and time points, with the exception of PP-C at 25 μg/mL (Figure 6D). Comparatively, at 3 h and an exposure concentration of 25 μg/mL, PEMA-B induced the most up-regulation of HMOX-1, while PS-P and PP-C induced the least. For macrophages exposed to 50 μg/mL of plastic particles for 3 h the PET-W induced the greatest up-regulation of HMOX-1. At 24 h, HDEP-M induced the least up-regulation of HMOX-1, compared to other plastic particles at both 25 and 50 μg/mL. At 25 μg/mL PEMA-B induced the greatest up-regulation of HMOX-1, compared to other plastic particles, while PET-W and PS-P induced the greatest up-regulation of HMOX-1 at 50 μg/mL. Exposure to pristine commercially available PS NP at 10 μg/mL was determined to induce significant reactive oxygen species generation in RAW 264.7 cells after a 6 h exposure [31]. This is consistent with the robust upregulation of HMOX-1 observed in RAW 264.7 cells exposed to our PS NPs for 24 h. Specifically, HMOX-1 is a vital antioxidant enzyme responsible for the cleavage of heme groups, and is induced via Nrf2 following exposure that causes inflammation and oxidative stress. Previous research has demonstrated pristine PS NPs can increase reactive oxygen species production and mitochondrial membrane damage, and has shown these effects are exacerbated following functionalization with -COOH or -NH_2_ [28]. 

At higher doses NP exposures may induce significant mitochondrial membrane damage and/or oxidative stress to cause the cytotoxicity observed in our study (Figure 6). The irregular shapes of the NPs generated in our current study may initiate more cellular damage and provide additional surface area for interactions, contributing to enhanced inflammatory and oxidative stress responses.

## 3. Materials and Methods

### 3.1. Materials

Various disposable plastic materials were collected from domestic garbage, including water bottles, styrofoam plates, milk bottles, centrifuge tubes, to-go food boxes, and white plastic bags. Coarse sea salt crystals (Sicilian Sea Salt, a product of Italy) were obtained from CENTO Fine Foods in Deptford, NJ, USA. A Ball Mill (PQ-N2, 0.4–4.0 L Planetary Ball Mill) was acquired from Across International in Livingston, NJ, USA. A 70 µm sieve was sourced from W.S.tyler in Mentor, OH, USA, and a 0.22 µm nylon membrane filter was used in the experiments.

For the biological aspects of the study, a mouse macrophage cell line (RAW 264.7) was obtained from ATCC in Manassas, VA, USA. Thiazolyl Blue Tetrazolium Bromide (MTT) cell proliferation assay kits were procured from Sigma Aldrich in St. Louis, MO, USA. Trizol reagent was acquired from Invitrogen in Carlsbad, CA, USA, and Direct-zolTM RNA MiniPrep Kits were sourced from Zymo Research in Irvine, CA, USA.

### 3.2. Microplastic and Nanoplastic Preparation

The plastic-based materials listed in Table 1 were collected randomly from the surrounding area. To prepare them for analysis, they underwent a thorough washing process using Milli-Q water to remove any impurities. Subsequently, they were dried overnight. Raman Spectroscopy was employed to identify and analyze the composition of these materials, and the results are provided in Table 1. To convert these plastic materials into microplastics (MPs), they were cut into small pieces and subjected to milling using a planetary Ball Mill (PQN2 Ball Mill, Across International, USA). The milling process involved utilizing coarse iodized salt as the milling media, with the mill operating at 580 RPM.

After milling, the ball mill canisters, which contained both the salt particles and the micronized plastic, were filled with Milli-Q water and then passed through a 70 μm sieve to eliminate any larger particles. The resulting solution, containing smaller-sized particles (including both salt and plastics), underwent high-speed stirring for 30 min using a magnetic stirrer to dissolve any remaining salt particles. The solution was then filtered through a 0.2 μm nylon filter for several hours to isolate the microplastics. Following the filtration step, the sample was washed multiple times with Milli-Q water to remove any remaining impurities, and the microplastics were left to dry at room temperature overnight until a constant dry weight was achieved. To ensure accurate characterization, several analytical techniques were employed to study the microplastic samples thoroughly.

To produce the NPs, the dried microplastic particles were re-dispersed in 50 mL of Milli-Q water. To break down larger particles and agglomerates into smaller ones, the suspension was subjected to sonication using a high-power ultrasonic probe sonicator, with 70% of the total wattage (800 watts) applied for approximately 5 min. After sonication, the suspension was allowed to cool, and the smaller-sized particles were sieved and separated. This separation process involved letting the suspended particles come to rest for a certain period, during which the upper layer containing the water-dispersed nanoparticles was collected for further analysis. This step helped isolate the desired nanoparticles from the rest of the suspension, ensuring a more precise characterization and study of the produced NPs.

### 3.3. Physical and Chemical Characterization

Particle size and zeta potential for all the microplastic and nanoplastic samples were characterized using Malvern Nano ZS Dynamic Light Scattering DLS, Nano ZS 90, Model: ZEN 3690, Worcestershire, UK). The surface imaging of the samples was carried out by Scanning Electron Microscope (SEM, JSM-7900 F, JEOL, Tokyo, Japan). The specimens were carbon coated for imaging using EMS 150 TES sputtering coater (Hatfield, PA, USA). The particles’ structural properties were identified by using a Fisher Scientific Raman Microscope, Hampton, NH, USA (DXR2xi 532 nm wavelength laser and filter) and by Fourier-transform infrared spectroscopy (FTIR) (Shimadzu, Kyoto, Japan, IRAffinity-1). 

### 3.4. Cell Culture and Exposure

A mouse macrophage cell line (RAW 264.7) (ATCC, Manassas, VA, USA) was utilized as a representative model to examine cellular responses to plastic particulates. Cells were cultured and maintained in culture petri dishes using Dulbecco’s modified Eagle’s medium supplemented with 10% fetal bovine serum and 1% antibiotic at 37C and 5% CO_2_, similar to our previous studies. Cells were seeded in either 24- or 96-well plates, as needed for experiments. After cells reached 90% confluency, they were exposed to plastic particulates for analysis of cell viability and alterations in gene expression.

### 3.5. Assessment of Cell Viability

RAW 264.7 cells were exposed to plastic particulates at concentrations of 0, 6.25, 12.5, 25, 50, 100, 200, 400, or 800 ug/mL for 3 or 24 h, and cytotoxicity was assessed via the Thiazolyl Blue Tetrazolium Bromide (MTT) cell proliferation assay (Sigma Aldrich, St. Louis, MO, USA). After exposure, cells were incubated with the MTT reagent at a concentration of 0.5 mg/mL for 3 h within the cell culture incubator (37 °C and 5% CO_2_). During the MTT incubation insoluble purple crystals form within viable cells, which were dissolved through addition of DMSO. The color change was measured by a plate reader (Molecular Devices, San Jose, CA, USA) at 570 nm. Exposure-induced alterations in viability were determined by comparison to the control group (*n* = 4/group). Three technical replicates were present on each plate and averaged together to create an individual sample. Untreated cells were used as controls for the MTT cell viability assay.

### 3.6. Evaluation of Inflammatory Gene Expression

Plastic particulate variation in inflammatory response following exposure were assessed via alterations in gene expression. Macrophages were exposed to 25 or 50 μg/mL of plastic particulates for 3 or 24 h. After the exposure, cells were collected in Trizol (Invitrogen, Carlsbad, CA, USA), and total RNA was isolated by Direct-zolTM RNA MiniPrep Kits (Zymo Research, Irvine, CA, USA), following the manufacturer’s instructions. After extraction of RNA, the concentration was measured via Nanodrop (Thermo Scientific, Hercules, CA, USA). A total amount of 1000 ng of RNA was reversely transcribed into cDNA using a cDNA synthesis kit (Bio-Rad, Hercules, CA, USA) in a thermal cycler (Eppendorf, Enfield, CT, USA). After cDNA synthesis, alterations in inflammatory gene expression markers, including Macrophage chemoattactant protein-1 (MCP-1), Macrophage inflammatory protein-1 (CXCL2), and Interleukin-6 (IL-6), as well as the oxidative stress marker Hemeoxygenase-1 (HMOX-1), were assessed utilizing mouse-specific primers (Qiagen, Hilden, Germany) through quantitative real-time reverse transcriptase polymerase chain reaction (real-time RT PCR). Glyceraldehyde 3-phosphate dehydrogenase (GAPDH) was used as the internal control for all gene expressions. Relative fold gene expression of samples among different groups was calculated by using the delta-delta CT method (*n* = 5/group). Untreated cells were utilized as controls and 2 technical replicates for each sample were used when running real-time RT PCR.

### 3.7. Statistical Analysis

All cell toxicity assessment results are shown as mean values ± S.E.M, with 4–5 samples per group (3 assay technical replicates for cytotoxicity and 2 assay technical replicates for gene expression). Comparisons of cell viability and gene expression alterations were statistically assessed using one-way ANOVAs with Tukey post hoc analysis between groups. Using exposure as the factor, *p* < 0.05 was considered to be statistically significant. All statistical analyses were processed in GraphPad Prism version 9 software (GraphPad, San Diego, CA, USA).

## 4. Conclusions

In conclusion, this study sheds light on the environmental and health impacts of NP, which have become increasingly prevalent in our daily lives. The majority of previous studies have relied on synthesized polystyrene, which typically has the same size and spherical shape. However, in this study, a novel top-down milling technique was used to create NP through abrasion with sea salt. This technique did not require solvents, cryogenics, or milling media. By synthesizing NP directly from waste plastics, this approach generated particles that were closer to real-world NP.

Our findings also highlight the toxic impact of the synthesized NP on cell lines, underscoring the urgent need for more research on this topic. The study examined the effect of different types of plastic particles on the inflammatory gene expression of a macrophage cell line. All plastic particles induced the production of macrophage chemoattractant protein-1 (MCP-1), macrophage inflammatory protein-2 (CXCL2), interleukin-6 (IL-6), and heme-oxygenase-1 (HMOX-1) markers of inflammation and oxidative stress. However, the degree of induction varied, depending on the type of plastic particle, concentration, and duration of exposure. Overall, this study contributes to our understanding of the environmental and health implications of microplastics and nanoplastics, and offers a potential solution for their safe and sustainable preparation.

## Figures and Tables

**Table 1:** Analysis of synthesized NP by Dynamic Light Scattering (DLS) technique.

Supplied Plastic	Polymer Ingredients	Samples	Particle Size [nm]	Poly Dispersity Index [PDI]	Nanoform Zeta Potential [mV]
Water Bottle	Polyethylene Terephthalate (PET)	PET-W	50.75	0.075	−28.6
Styrofoam Plate	Polystyrene (PS)	PS-P	396.1	0.259	−28.5
Milk Gallon	High Density Polyethylene (HDPE)	HDPE-M	141.8	0.558	−32.1
Centrifuge Tube	Polypropylene (PP)	PP-C	255.0	0.883	−29.4
To Go Black Box	Poly (Ethylene-co-Methacrylic Acid) (PEMA)	PEMA-5	190.1	0.961	−22.4

## Data Availability

Data are contained within the article and Supplementary Materials.

## Supplementary Materials

The following supporting information can be downloaded at: https://www.mdpi.com/article/10.3390/molecules29092033/s1.
