# Supplementary material for: Generation of Eroded Nanoplastics from Domestic Wastes and Their Impact on Macrophage Cell Viability and Gene Expression
Source: Molecules. 2024 Apr 28;29(9):2033. doi: 10.3390/molecules29092033 (PMC11085467; doi:10.3390/molecules29092033)
Supplement: Supplementary file 1 [file molecules-29-02033-s001.zip › molecules-2937167-supplementary.pdf]

Supplementary data

The crystallite size is an important parameter as the sizes of the crystals determine whether the material is soft (small crystallites) or brittle. Crystallite size determined via XRD corresponds to the volume of material for the respective diffraction peak which corresponds to the grain size or thickness of nanoplastic.

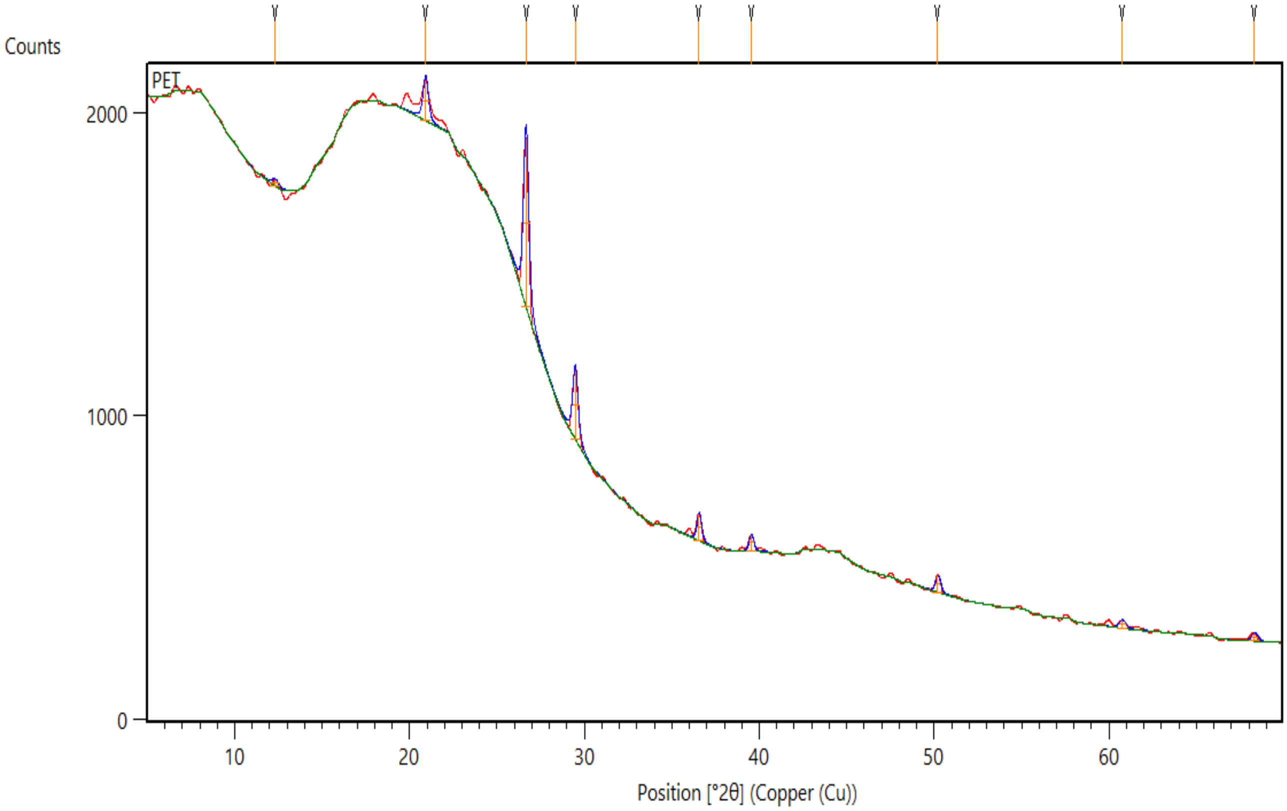

Peak List

Quantification | Anchor Scan Data | Smart Batches | PDF Analysis | Distances and Angles | Pattern List | Peak List X | Scan List | Structure Plot

No.	Pos. [°2θ]	d-spacing ...	Height [c...	FWHM Left ...	F...	Sha...	S...	Integral Breadt...	Area [cts*°2θ]	h	k	l	M...	Backgr.[cts]	St...	Crystallite Size only [Å]	Micro Strain onl...	Crystallite Size [Å]	Micro Strain [%]
1	12.3238	7.17635	21.07	0.4861		0.600		0.47951	10.10					1758.34	In...	187	1.92046	-1164	1.92025
2	20.9054	4.24586	135.59	0.3454		0.600		0.34070	46.19					1974.69	In...	268	0.79175	-1197	0.79166
3	26.6534	3.34182	551.76	0.3326		0.600		0.32809	181.02					1362.97	In...	282	0.59312	-1220	0.59306
4	29.4687	3.02865	225.95	0.3326		0.600		0.32809	74.13					924.80	In...	284	0.53431	-1231	0.53425
5	36.5444	2.45684	89.37	0.2814		0.600		0.27761	24.81					588.61	In...	344	0.35744	-1258	0.35740
6	39.5223	2.27832	53.37	0.2814		0.600		0.27761	14.82					553.37	In...	347	0.32851	-1270	0.32848
7	50.1945	1.81607	56.27	0.3070		0.600		0.30285	17.04					418.07	In...	329	0.27595	-1308	0.27592
8	60.7354	1.52369	27.16	0.4093		0.600		0.40380	10.97					299.95	In...	257	0.29685	-1341	0.29681
9	68.2931	1.37232	28.87	0.3454		0.600		0.34070	9.84					257.45	In...	319	0.21499	-1360	0.21497

Figure S1: PET-W sample crystallite size determination by XRD HighScore Plus software

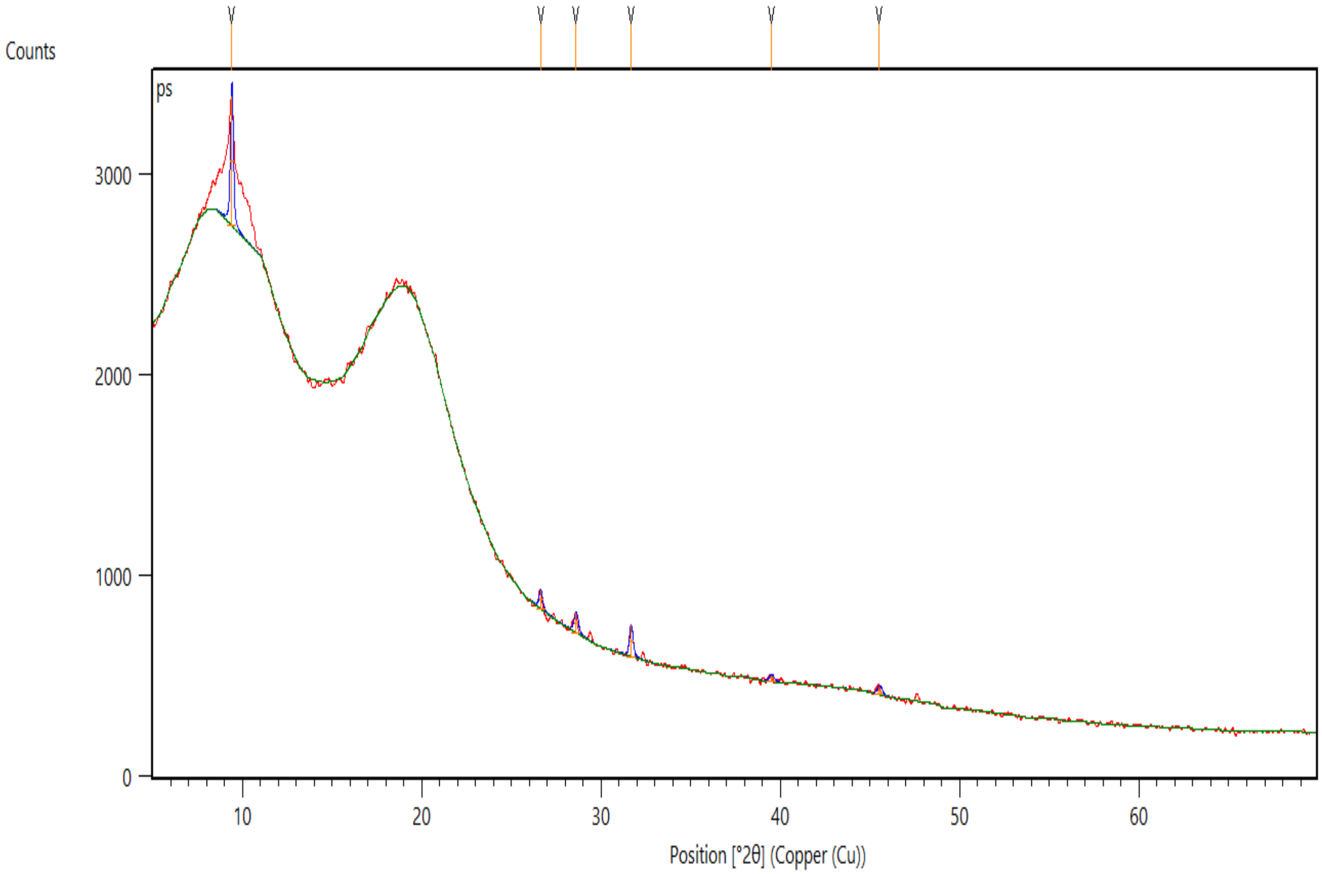

Peak List

Quantification | Anchor Scan Data | Smart Batches | PDF Analysis | Distances and Angles | Pattern List | Peak List | Scan List | Structure Plot

No.	Pos. [°2θ]	d-spacing ...	Height [c...	FWHM Left ...	FWHM Rig...	Sha...	Shape Ri...	Integral Breadt...	Area [cts*°2θ]	h	k	l	Multipli...	Backgr.[cts]	Status	Crystallite Size only [Å]	Micro Strain onl...	Crystallite Size [Å]	Micro Strain [%]
1	9.4208	9.38023	650.05	0.1791		0.600		0.17666	114.84					2740.05	Incl...	540	0.86972	-1153	0.86962
2	26.6303	3.34466	91.81	0.2047		0.600		0.20190	18.54					833.01	Incl...	473	0.35364	-1219	0.35360
3	28.5998	3.11865	99.12	0.2558		0.600		0.25237	25.01					713.27	Incl...	373	0.41841	-1227	0.41837
4	31.6689	2.82307	153.82	0.2047		0.600		0.20190	31.06					594.53	Incl...	478	0.29525	-1239	0.29521
5	39.4924	2.27997	35.53	0.2814		0.600		0.27761	9.86					468.72	Incl...	347	0.32878	-1270	0.32875
6	45.5098	1.99152	45.11	0.2814		0.600		0.27761	12.52					404.02	Incl...	354	0.28133	-1292	0.28130

Figure S2: PS-P sample crystallite size determination by XRD HighScore Plus software

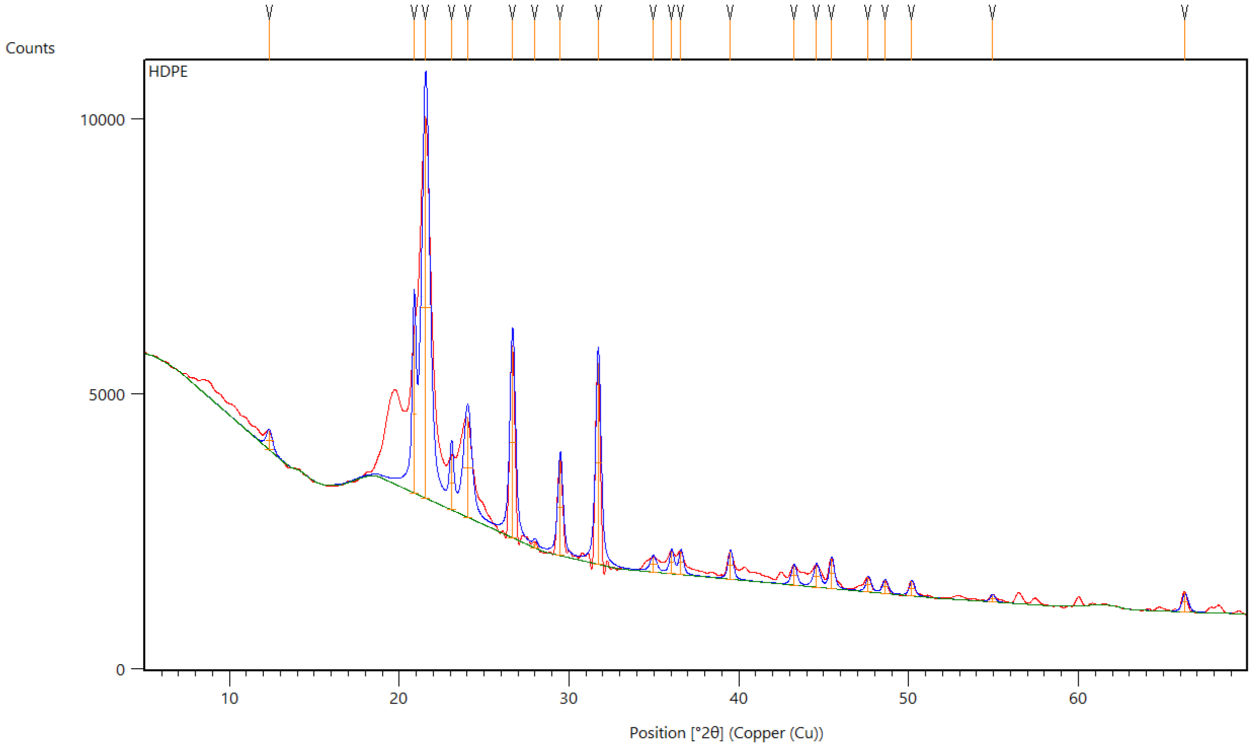

Peak List

Quantification | Anchor Scan Data | Smart Batches | PDF Analysis | Distances and Angles | Pattern List | Peak List | Scan List | Structure Plot

No.	Pos. [°2θ]	d-spacing ...	Height [c...	FWHM Left ...	FWHM Rig...	Sha...	Shape Ri...	Integral Breadt...	Area [cts*°2θ]	h	k	l	Multipli...	Backgr.[cts]	Status	Crystallite Size only [Å]	Micro Strain onl...	Crystallite Size [Å]	Micro Strain [%]
1	12.3504	7.16099	336.95	0.4221		0.600		0.41642	140.31					3976.76	Incl...	216	1.65913	-1164	1.65894
2	20.8695	4.25307	2884.12	0.2303		0.600		0.22714	655.09					3196.37	Incl...	412	0.51669	-1197	0.51664
3	21.5509	4.12011	6925.05	0.5373		0.600		0.52998	3670.17					3100.55	Incl...	171	1.20645	-1199	1.20632
4	23.0773	3.85094	993.38	0.2558		0.600		0.25237	250.70					2885.90	Incl...	369	0.52217	-1205	0.52211
5	24.0215	3.70166	1795.77	0.5373		0.600		0.52998	951.73					2753.11	Incl...	172	1.07928	-1209	1.07916
6	26.6644	3.34046	3484.53	0.3582		0.600		0.35332	1231.16					2381.44	Incl...	261	0.64016	-1220	0.64009
7	27.9931	3.18485	115.06	0.2558		0.600		0.25237	29.04					2194.59	Incl...	373	0.42785	-1225	0.42780
8	29.4737	3.02814	1725.34	0.3326		0.600		0.32809	566.06					2054.76	Incl...	284	0.53421	-1231	0.53415
9	31.7035	2.82007	3663.74	0.3454		0.600		0.34070	1248.25					1897.65	Incl...	274	0.51464	-1239	0.51459
10	34.9504	2.56516	267.38	0.4221		0.600		0.41642	111.34					1755.17	Incl...	225	0.57063	-1252	0.57056
11	36.0323	2.49058	418.27	0.2814		0.600		0.27761	116.12					1724.29	Incl...	343	0.36288	-1256	0.36284
12	36.5798	2.45455	422.74	0.2942		0.600		0.29023	122.69					1708.66	Incl...	328	0.37415	-1258	0.37411
13	39.4942	2.27987	512.11	0.3198		0.600		0.31547	161.55					1625.47	Incl...	304	0.37585	-1270	0.37581
14	43.2331	2.09097	364.16	0.3326		0.600		0.32809	119.48					1518.76	Incl...	295	0.35464	-1284	0.35460
15	44.5601	2.03173	398.79	0.3966		0.600		0.39118	156.00					1480.88	Incl...	247	0.41123	-1288	0.41118
16	45.4519	1.99392	550.27	0.3198		0.600		0.31547	173.59					1455.43	Incl...	310	0.32209	-1292	0.32206
17	47.6158	1.90823	276.54	0.3070		0.600		0.30285	83.75					1393.66	Incl...	326	0.29299	-1299	0.29295
18	48.6002	1.87186	247.46	0.3326		0.600		0.32809	81.19					1365.56	Incl...	301	0.31118	-1303	0.31114
19	50.1750	1.81673	281.10	0.3198		0.600		0.31547	88.68					1321.52	Incl...	316	0.28809	-1308	0.28806
20	54.9221	1.67040	138.81	0.3070		0.600		0.30285	42.04					1213.40	Incl...	336	0.24860	-1324	0.24857
21	66.2421	1.40975	364.67	0.3326		0.600		0.32809	119.64					1030.77	Incl...	328	0.21497	-1355	0.21494

Figure S3: HDPE-M sample crystallite size determination by XRD HighScore Plus software

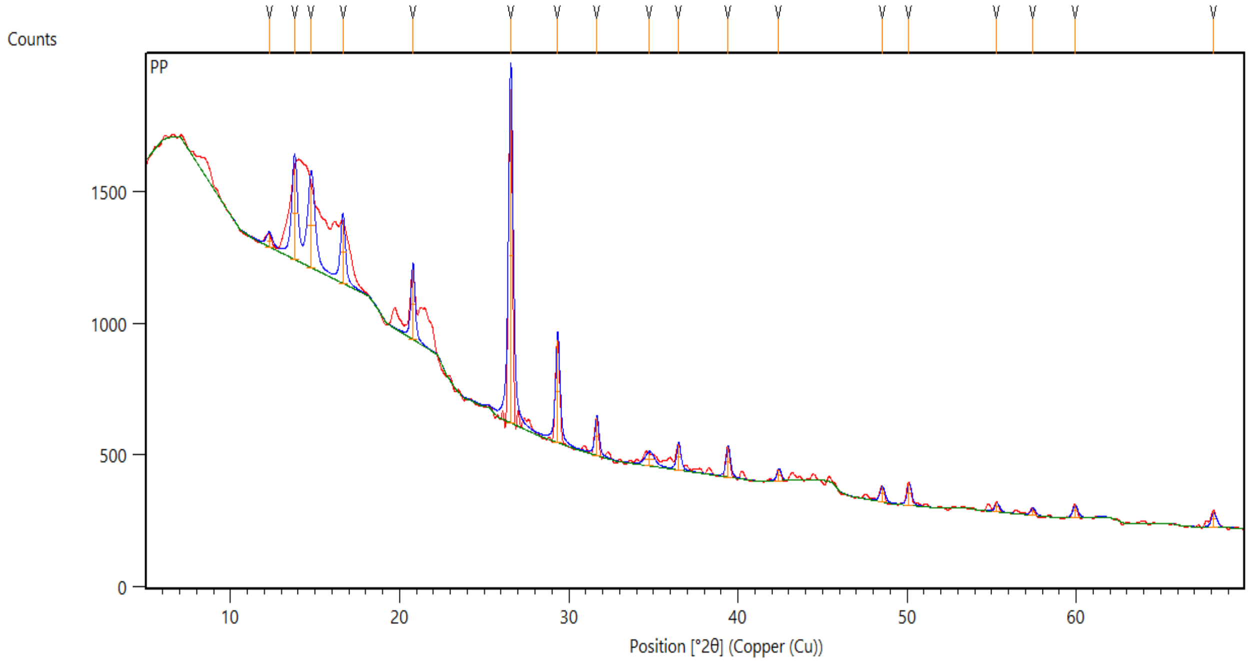

Peak List

Quantification | Anchor Scan Data | Smart Batches | PDF Analysis | Distances and Angles | Pattern List | Peak List | Scan List | Structure Plot

No.	Pos. [°2θ]	d-spacing ...	Height [c...	FWHM Left ...	FWHM Rig...	Sha...	Shape Ri...	Integral Breadt...	Area [cts*°2θ]	h	k	l	Multipli...	Backgr.[cts]	Status	Crystallite Size only [Å]	Micro Strain onl...	Crystallite Size [Å]	Micro Strain [%]
1	12.2915	7.19514	47.39	0.3838		0.600		0.37856	17.94					1290.88	Incl...	238	1.51163	-1164	1.51146
2	13.7852	6.41872	351.34	0.3710		0.600		0.36594	128.57					1243.30	Incl...	247	1.30039	-1169	1.30024
3	14.7723	5.99192	322.43	0.4733		0.600		0.46689	150.54					1211.85	Incl...	193	1.55671	-1173	1.55654
4	16.6330	5.32560	237.15	0.3070		0.600		0.30285	71.82					1152.57	Incl...	302	0.88362	-1180	0.88352
5	20.7763	4.27194	269.64	0.2558		0.600		0.25237	68.05					938.94	Incl...	368	0.58137	-1196	0.58131
6	26.5564	3.35380	1269.81	0.2942		0.600		0.29023	368.54					621.96	Incl...	320	0.52386	-1219	0.52380
7	29.3294	3.04271	391.74	0.2814		0.600		0.27761	108.75					546.85	Incl...	338	0.45090	-1230	0.45085
8	31.6478	2.82491	144.76	0.2558		0.600		0.25237	36.53					498.29	Incl...	376	0.37636	-1239	0.37632
9	34.7657	2.57836	50.16	0.6140		0.600		0.60570	30.38					458.14	Incl...	154	0.83978	-1251	0.83969
10	36.4823	2.46089	101.64	0.2558		0.600		0.25237	25.65					442.48	Incl...	380	0.32370	-1258	0.32366
11	39.4017	2.28501	119.61	0.2558		0.600		0.25237	30.19					415.83	Incl...	384	0.29795	-1269	0.29791
12	42.4059	2.12982	46.09	0.2558		0.600		0.25237	11.63					401.99	Incl...	388	0.27499	-1280	0.27496
13	48.5157	1.87492	59.40	0.2942		0.600		0.29023	17.24					321.17	Incl...	342	0.27434	-1303	0.27431
14	50.1040	1.81914	89.32	0.2558		0.600		0.25237	22.54					308.32	Incl...	399	0.22812	-1308	0.22809
15	55.2961	1.65999	37.04	0.2430		0.600		0.23975	8.88					283.57	Incl...	431	0.19253	-1325	0.19251
16	57.4145	1.60367	29.40	0.2558		0.600		0.25237	7.42					270.54	Incl...	413	0.19451	-1332	0.19448
17	59.9040	1.54284	50.87	0.2942		0.600		0.29023	14.76					260.10	Incl...	360	0.21429	-1339	0.21427
18	68.1000	1.37574	64.70	0.2686		0.600		0.26499	17.15					224.08	Incl...	416	0.16565	-1359	0.16563

Figure S4: PP-C sample crystallite size determination by XRD HighScore Plus software

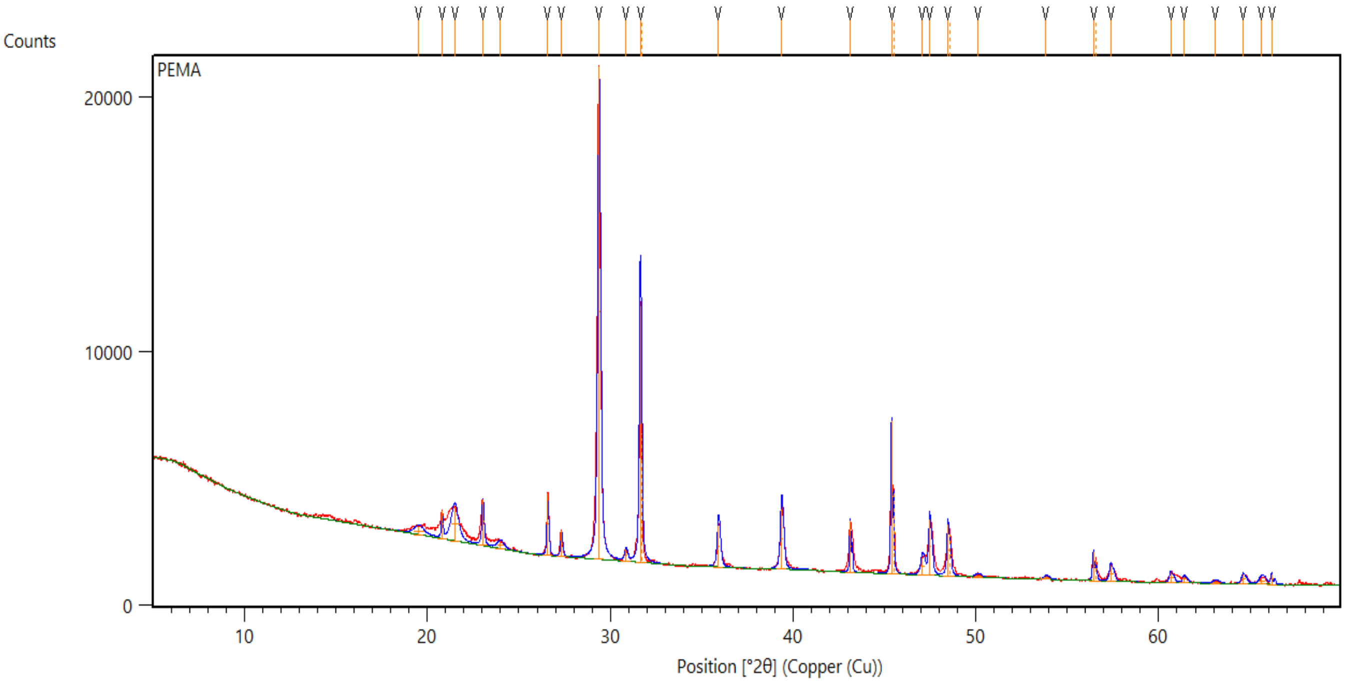

Peak List

Quantification | Anchor Scan Data | Smart Batches | PDF Analysis | Distances and Angles | Pattern List | Peak List | Scan List | Structure Plot

No.	Pos. [°2θ]	d-spacing ...	Height [c...	FWHM Left ...	FWHM Rig...	Sha...	Shape Ri...	Integral Breadt...	Area [cts*°2θ]	h	k	l	Multipli...	Backgr.[cts]	Status	Crystallite Size only [Å]	Micro Strain onl...	Crystallite Size [Å]	Micro Strain [%]
1	19.5325	4.54108	340.65	0.6140		0.600		0.60570	206.33					2767.97	Incl...	149	1.52709	-1192	1.52692
2	20.7989	4.26737	1113.39	0.0768		0.600		0.07571	84.30					2610.87	Incl...	2185	0.09771	-1197	0.09770
3	21.4810	4.13337	1340.63	0.4605		0.600		0.45427	609.01					2526.25	Incl...	200	1.03482	-1199	1.03470
4	23.0226	3.85996	1846.00	0.1279		0.600		0.12619	232.94					2334.99	Incl...	826	0.23382	-1205	0.23379
5	23.9951	3.70568	300.53	0.4093		0.600		0.40380	121.35					2214.35	Incl...	226	0.81893	-1209	0.81884
6	26.5706	3.35204	2464.97	0.1023		0.600		0.10095	248.84					1949.11	Incl...	1152	0.14567	-1219	0.14565
7	27.3331	3.26024	1032.43	0.1151		0.600		0.11357	117.25					1907.76	Incl...	962	0.16956	-1222	0.16954
8	29.3766	3.03793	19451.52	0.1663		0.600		0.16404	3190.88					1796.95	Incl...	603	0.25223	-1230	0.25220
9	30.8583	2.89535	474.09	0.1535		0.600		0.15142	71.79					1716.60	Incl...	665	0.21785	-1236	0.21783
10	31.6366	2.82588	10124.71	0.1248		0.600		0.16640	1684.75					1674.40	Incl...	1089	0.12973	2548	0.09123
11	31.7421	2.81673	4894.50	0.0468		0.600		0.06240	305.42					1668.68	Incl...	29088	0.00485	-2804	-0.08254
12	35.9176	2.49827	1629.28	0.1716		0.600		0.22880	372.78					1492.62	Incl...	596	0.20953	1198	0.13569
13	39.3714	2.28670	2370.72	0.1716		0.600		0.22880	542.42					1399.73	Incl...	602	0.18985	1210	0.12294
14	43.1184	2.09627	2056.42	0.0780		0.600		0.10400	213.87					1282.55	Incl...	30010	0.00349	-18206	-0.03533
15	45.3818	1.99684	6067.90	0.0780		0.600		0.10400	631.06					1220.60	Incl...	30252	0.00330	-17945	-0.03359
16	45.5115	1.99145	3231.84	0.0780		0.600		0.10400	336.11					1217.05	Incl...	30341	0.00329	-17970	-0.03350
17	47.0715	1.92902	682.28	0.2184		0.600		0.29120	198.68					1174.35	Incl...	434	0.22248	813	0.13756
18	47.4754	1.91355	2237.03	0.1404		0.600		0.18720	418.77					1163.30	Incl...	888	0.10778	1945	0.07336
19	48.4753	1.87639	2052.36	0.1404		0.600		0.18720	384.20					1135.93	Incl...	892	0.10516	1956	0.07159
20	48.6060	1.87165	1664.22	0.0936		0.600		0.12480	207.70					1132.36	Incl...	4763	0.01970	11871	0.01425
21	50.1078	1.81901	102.84	0.3744		0.600		0.49920	51.34					1091.25	Incl...	223	0.40804	381	0.23439
22	53.8309	1.70166	135.16	0.2496		0.600		0.33280	44.98					1007.56	Incl...	374	0.22766	682	0.13794
23	56.4283	1.62934	1158.55	0.1092		0.600		0.14560	168.68					950.35	Incl...	1837	0.04435	4797	0.03270
24	56.5674	1.62566	904.39	0.0780		0.600		0.10400	94.06					947.49	Incl...	31773	0.00256	-15062	-0.02786
25	57.3862	1.60440	657.61	0.1872		0.600		0.24960	164.14					930.59	Incl...	571	0.14039	1124	0.08968
26	60.6597	1.52541	411.58	0.1872		0.600		0.24960	102.73					874.53	Incl...	584	0.13070	1151	0.08365
27	61.4005	1.50877	271.36	0.1872		0.600		0.24960	67.73					864.96	Incl...	587	0.12862	1158	0.08236
28	63.0993	1.47218	105.34	0.3744		0.600		0.49920	52.58					843.00	Incl...	238	0.30896	409	0.17808
29	64.6187	1.44118	374.21	0.2184		0.600		0.29120	108.97					823.36	Incl...	478	0.15076	906	0.09389
30	65.6401	1.42121	277.21	0.3744		0.600		0.49920	138.38					810.15	Incl...	242	0.29364	416	0.16943
31	66.1785	1.41095	430.36	0.0936		0.600		0.12480	53.71					803.19	Incl...	15591	0.00452	22161	-0.00838

Figure S5: PEMA-5 sample crystallite size determination by XRD HighScore Plus software.
